# The integration site of the *APP *transgene in the J20 mouse model of Alzheimer’s disease

**DOI:** 10.12688/wellcomeopenres.12237.2

**Published:** 2018-10-10

**Authors:** Justin L. Tosh, Matthew Rickman, Ellie Rhymes, Frances E. Norona, Emma Clayton, Lennart Mucke, Adrian M. Isaacs, Elizabeth M.C. Fisher, Frances K. Wiseman

**Affiliations:** 1 Department of Neurodegenerative Disease, Institute of Neurology, University College London, London, WC1N 3BG, UK; 2 Gladstone Institute of Neurological Disease and University of California, San Francisco, CA, 4158, USA; 3 UK Dementia Research Institute, University College London, London, WC1E 6BT, UK

**Keywords:** Transgenic, mouse model, Alzheimer’s disease, APP, Zbtb20, J20, Amyloid Precursor Protein

## Abstract

**Background: **Transgenic animal models are a widely used and powerful tool to investigate human disease and develop therapeutic interventions. Making a transgenic mouse involves random integration of exogenous DNA into the host genome that can have the effect of disrupting endogenous gene expression. The J20 mouse model of Alzheimer’s disease (AD) is a transgenic overexpresser of human APP with familial AD mutations and has been extensively utilised in preclinical studies and our aim was to determine the genomic location of the J20 transgene insertion.

**Methods: **We used a combination of breeding strategy and Targeted Locus Amplification with deep sequencing to identify the insertion site of the J20 transgene array. To assess RNA and protein expression of *Zbtb20,* we used qRT-PCR and Western Blotting.

**Results:** We demonstrate that the J20 transgene construct has inserted within the genetic locus of endogenous mouse gene *Zbtb20 *on **chromosome 16 in an array *, *disrupting expression of **mRNA from this gene in adult hippocampal tissue. Preliminary data suggests that ZBTB20 protein levels remain unchanged in this tissue, however further study is necessary. We note that the endogenous mouse *App* gene also lies on chromosome 16, although 42 Mb from the *Zbtb20 *locus.

**Conclusions:** These data will be useful for future studies utilising this popular model of AD, particularly those investigating gene interactions between the J20 *APP *transgene and other genes present on Mmu16 in the mouse.

## Introduction

The Tg(PDGFB-APPSwInd)20Lms (MGI:3057148, here referred to as ‘J20’) mouse model is a transgenic animal that overexpresses mutant human APP protein (amyloid precursor protein), and is widely used as a model of amyloid deposition and pathogenesis in the study of Alzheimer’s disease (AD). J20 mice recapitulate many AD-like phenotypes, including synaptic loss, amyloid plaque deposition and cognitive impairment ( Hong *et al.*, 2016; Mucke *et al.*, 2000; Palop & Mucke, 2016).

The model was developed by Mucke and colleagues using the PDGF-APPSw,Ind transgene construct described previously ( Games *et al.*, 1995; Rockenstein *et al.*, 1995), which includes a human *APP* mini-gene, carrying the familial AD-linked 717 _V-F_ (Indiana) mutation ( Murrell *et al.*, 1991) and 670/671 _KM-NL_ (Swedish) double mutation ( Mullan *et al.*, 1992). The transgene construct was designed so that the *APP* mini-gene included genomic sequence for *APP* introns 6–8, allowing expression of hAPP695, hAPP751 and hAPP770 isoforms. The PDGF-APPSw,Ind transgene expression is driven in neurons throughout the brain by the human platelet-derived growth factor β chain (PDGFβ) promoter ( Harris *et al.*, 2010; Sasahara *et al.*, 1991). The J20 mouse is an important model: currently, 125 articles have been catalogued in the Mouse Genome Database bibliography ( Blake *et al.*, 2017), which report genotypic and/or phenotypic data from this mouse. This strain has been used for several classical genetic studies to determine the interaction of genes of interest with the *APP* transgene including a seminal report of the importance of Tau to Aß-associated neuronal dysfunction ( Roberson *et al.*, 2007). Moreover, this model has been used to elucidate the role of Aß in synaptic dysfunction ( Palop & Mucke, 2016; Palop *et al.*, 2007; Sanchez *et al.*, 2012).

Transgenic mice are conventionally generated by direct injection of linear foreign DNA into the pronucleus of fertilised zygotes. Once inside the cell, these linear fragments undergo circularisation and concatemer formation before integrating into the host genome as a tandem array ( Bishop & Smith, 1989). In principle, transgenes insert randomly into the host genome; however, ∼45% of integration sites lie within host gene regions (∼13.2 exonic, 31.6% intronic), potentially as a result of increased accessibility of transcriptionally active DNA ( Yan *et al.*, 2013). Integration of a transgene array into coding sequences can induce new mutations (for example, haploinsufficiency) ( Haruyama *et al.*, 2009), and so it is important to know the site of integration for a transgene array in a mouse model.

A recent study suggested an association of a heterozygous deletion of the *CHMP2B* gene (charged multivesicular body protein 2B) with early-onset Alzheimer’s disease ( Hooli *et al.*, 2014). Interestingly, mutations in *CHMP2B* are a rare genetic cause of Frontotemporal dementia ( Skibinski *et al.*, 2005). We have previously reported generation of a *Chmp2b* knockout mouse ( Ghazi-Noori *et al.*, 2012). To determine if *Chmp2b* deletion modulates APP/Aß biology *in vivo*, we attempted to cross our *Chmp2b* knockout with the J20 mouse, to study potential double mutant progeny. The *Chmp2b* locus lies on mouse chromosome 16.

Here, we present the outcome of these genetic cross experiments and the resulting mapping of the J20 transgene array integration site by Targeted Locus Amplification (TLA) with deep sequencing. We discuss how integration of the transgene affects expression of the flanking loci.

## Methods

### Animal welfare

Mice were housed in controlled conditions in accordance with guidelines from the UK Medical Research Council in Responsibility in Use of Animals for Medical Research (1993). Two female J20 positive animals were killed at 6 months of age to provide splenic material for the TLA study. Furthermore, 3 month hippocampal tissue was collected from J20 animals: N=5, 3 male, 2 female. C57BL/6J controls: N=5, 3 male, 2 female. All used for qRT-PCR and western blotting. All experiments were conducted under license from the UK Home Office and with Local Ethical Review approval. Tg(PDGFB-APPSwInd)20Lms/2Mmjax animals (J20) were obtained from The Jackson laboratory (stock no. 034836) and maintained on a C57BL/6J background in our animal facility. Chmp2b knockout animals were already available in our animal facility ( Ghazi-Noori *et al.*, 2012). Mice had access to a mouse house with bedding material and wood chips. All animals had continual access to water and RM1 (Special Diet Services) (stock animals) or RM3 (Special Diet Services) (breeding animals) chow *ad libitum*. Mice were housed in individually ventilated cages in a specific pathogen free facility with a 12 hour light/dark cycle.

### Genotyping of J20 and *Chmp2b* knockout mice

DNA was extracted from tail tip or ear biopsy by the HOTSHOT method ( Truett *et al.*, 2000).

The presence of the J20 human APP transgene was tested by PCR using primers (Eurofins) *APP*-F: 5’-GTGAGTTTGTAAGTGATGCC-3’ *APP*-R: 5’-TCTTCTTCTTCCACCTCAGC-3’, control primers ContF: 5’-CAAATGTTGCTTGTCTGGTG-3’ ContR: 5’-GTCAGTCGAGTGCACAGTTT-3). Copy number of the human APP transgene was validated by quantitative PCR using a Taqman Fast machine (Applied Biosystems) with the following primers and probes: h *APP*F: 5’-TGGGTTCAAACAAAGGTGCAA-3’ h *APP*R: 5’-GATGAAGATCACTGTCGCTATGAC-3’ hAPPprobe: FAM-CATTGGACTCATGGTGGGCGGTG-3’ qContF: 5’-CACGTGGGCTCCAGCATT-3’ qContR: 5’-TCACCAGTCATTTCTGCCTTTG-3’ qContProbe: VIC-CCAATGGTCGGGCACTGCTCAA-3’.

The *Chmp2b* knockout locus was detected by PCR with the following primers: Int2_F: 5’-CCATTGCCACTTGGATGTAA-3’ Int2_R: 5’-GACGCACTTTAAGGTCACAGC-3’ KO_R: 5’- TCTCTGTGCAAGAAGCATGAA-3’. PCR products were separated by agarose gel electrophoresis and visualised using a BioRad Gel Doc XR UV transilluminator.

### Extraction of spleen cells from J20 animals

Mice were sacrificed by rising concentration of CO _2_ and confirmed by dislocation of the neck and the spleen dissected and kept on ice. Splenocytes were then dissociated through a 40µm mesh filter and the cells collected by centrifugation at 4°C at 500 × *g* for 5 minutes. The supernatant was discarded and the pellet re-suspended in 1 ml red blood cell lysis buffer (4.13g NH _4_Cl, 0.5g KHCO _3_, 193.5µl 0.5M EDTA dissolved in 500ml H _2_O) for three minutes at room temperature to lyse splenic erythrocytes. To terminate the lysis reaction, 0.5ml phosphate buffered saline (PBS) was added and the splenocytes were collected again by centrifugation at 500 × g for 5 minutes. The supernatant was discarded and the pellet re-suspended in 0.5ml PBS before a final centrifugation step for 2 minutes. The supernatant was discarded and the pellet was re-suspended in 1ml freezing buffer (PBS with 10% fetal calf serum and 10% dimethyl sulphoxide). Samples were stored at -80°C before preparation for TLA processing.

### Targeted Locus Amplification

Processing of samples for TLA was performed by Cergentis B.V. (Utrecht, The Netherlands), as previously described ( de Vree *et al.*, 2014). A primer pair targeted to the *APP* transgene sequence was used to perform the TLA. Sequences of the PCR primers are (5’ to 3’): 1917_APP_F GAAACTCATCTTCACTGGCA; 1698_APP_R GGGTAGACTTCTTGGCAATA. PCR products were purified and library prepped using the Illumina NexteraXT protocol and sequenced on an Illumina Miniseq sequencer.

### Sequence alignment and analysis of TLA

TLA reads were mapped using BWA-SW, which is a Smith-Waterman alignment tool. This allows for partial mapping, which is optimally suited for identifying break-spanning reads. The mouse Mm10 genome assembly version was used for mapping. Visualisation and interpretation of the data were performed using the Integrative Genomics Viewer (IGV) from the Broad Institute ( Robinson *et al.*, 2011).

### RNA extraction and quantitative reverse transcription PCR

RNA was extracted from whole hippocampus from J20 animals and age and litter matched controls. Total RNA was extracted using the Qiagen miRNeasy kit and reverse transcribed using the Applied Biosystems High-Capacity RNA-to-cDNA™ Kit.

Quantitative RT-PCR was carried out on the *Zbtb20* gene transcript using a predesigned PrimeTime ^®^ probe-based qPCR assay (assay ID: Mm.PT.58.41805451, Integrated DNA Technologies [IDT]) targeted to exons 8–9 (RefSeq transcript NM_181058) with a FAM probe. TaqMan reactions were run with Taqman Universal Master Mix 2 on a 7500 Fast machine (Applied Biosystems) using standard cycling conditions. Transcript levels were normalised against Applied Biosystems mouse *Actb* (Assay ID: 4352933E) and Integrated DNA Technologies *B2m* (assay ID: Mm.PT.39a.22214835) endogenous controls in independent experiments and the results averaged geometrically. Both controls contained VIC probes.

### Tissue preparation and western blotting for ZBTB20

For analysis of ZBTB20 in hippocampus, J20 and age/sex-matched wildtype littermate controls were dissected under ice-cold PBS before homogenisation in radioimmunoprecipitation buffer (150mM sodium chloride, 50mM Tris, 1% NP-40, 0.5% sodium deoxycholate, 0.1% sodium dodecyl sulphate) with Protease Inhibitor Cocktail Set 1 (Merck). Total protein concentration was determined using Bradford assay (Bio-Rad). Samples from individual animals were run separately and not pooled.

Equal amounts of hippocampal brain proteins were denatured in LDS sample buffer (ThermoFisher) and β-Mercaptoethanol for 10 minutes at 100°C, prior to separation by SDS polyacrylamide gel electrophoresis in 4–12% pre-cast gels (ThermoFisher). Separated proteins were transferred to 0.2µm nitrocellulose membrane and blocked in 5% milk/phosphate buffered saline (with 0.05% Tween 20, PBST) for one hour at room temperature. The membrane was then cut horizontally at the 49KDa band (SeeBlue Plus II protein ladder, Invitrogen) and the lower half was incubated in mouse monoclonal antibody to β-Actin (A5441, Sigma-Aldrich) diluted 1:200,000 in 1% bovine serum albumin (BSA)/PBST overnight at 4°C. The upper half was incubated overnight with rabbit polyclonal primary antibody against ZBTB20 (23987-1-AP, ProteinTech) diluted 1:1000 in 1% BSA/PBST. After washing with PBST the upper and lower membranes were incubated with HRP-conjugated secondary rabbit and mouse antibodies, respectively, diluted in 1% BSA/PBST for 1 hour at room temperature. SuperSignal™ West Pico Chemiluminescent Substrate and X-ray film was used to visualise bands, Image J 1.49c software (NIH) was used to analyse band intensity. Graphpad prism 5 (Graphpad Software, Inc.) was used to plot graphs and SPSS 25 (IBMCorp) was used to perform statistical analyses.

## Results

### Mouse breeding strategy

**Figure 1.**
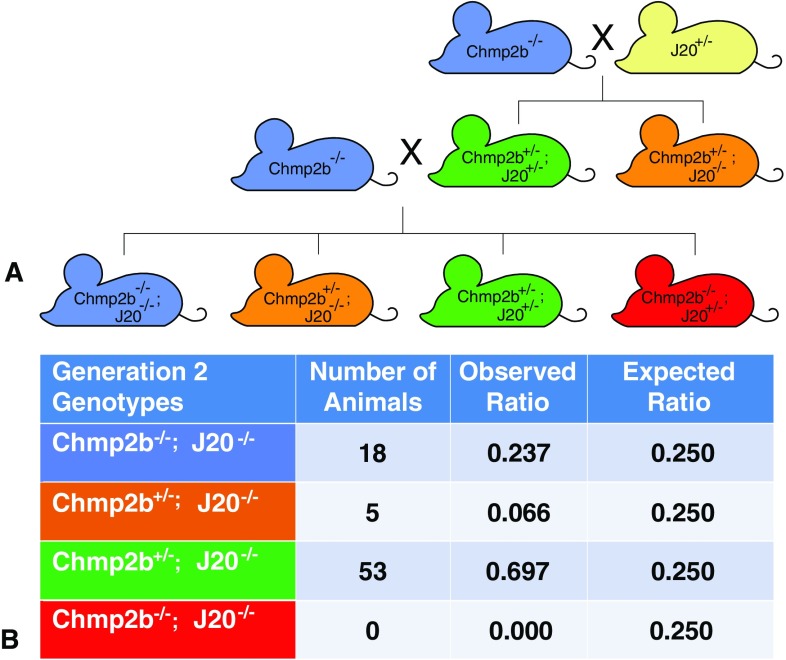
Breeding scheme and observed ratios of offspring in a two-generation cross between *Chmp2b* ^-/-^ and J20 ^+/-^ animals. ( **A**) Two-generation breeding scheme to generate Chmp2b ^-/-^; J20 ^+/-^ animals. ( **B**) Observed ratios of offspring genotype differed significantly from the expected ratio χ2(3, N = 90.21) = 0.58, p < 0.0001.

To investigate the role of *Chmp2b* in the pathogenesis of AD, we attempted to generate a homozygous knockout of *Chmp2b* in the Tg(PDGFB-APPSwInd)20Lms (J20) *APP* transgenic mouse strain. To accomplish this we set up matings over two generations ( Figure 1a), firstly between *Chmp2b ^-/-^* and hemizygous J20 mice (referred for simplicity as TgAPP ^J20/-^, where ‘J20’ denotes the presence of the transgene). This cross produced *Chmp2b* ^+/-^;Tg *APP* ^J20/-^ and *Chmp2b* ^+/-^;Tg *APP* ^-/-^ progeny. In the second stage, the *Chmp2b* ^+/-^;Tg *APP* ^J20/-^ offspring were crossed to *Chmp2b* ^-/-^ null animals. This cross was predicted to produce four progeny genotypes at equal Mendelian ratios (0.25):

(i) homozygous for *Chmp2b* knockout, without the *APP* transgene ( *Chmp2b* ^-/-^;Tg *APP* ^-/-^),
(ii) homozygous for *Chmp2b* knockout, hemizygous for *APP* transgene ( *Chmp2b* ^-/-^;Tg *APP* ^J20/-^),
(iii) hemizygous for *Chmp2b* knockout, hemizygous for *APP* transgene ( *Chmp2b* ^+/-^;Tg *APP* ^J20/-^),
(iv) hemizygous for *Chmp2b* knockout, without the *APP* transgene ( *Chmp2b* ^+/-^;Tg *APP* ^-/-^).

The second stage cross resulted in 76 progeny, but the observed ratio of genotypes significantly differed from the expected ratio ( Figure 1b). Strikingly no *Chmp2b* ^-/-^;Tg *APP* ^J20/-^ progeny were produced. This suggests that either *Chmp2b* ^-/-^;Tg *APP* ^J20^ may not be viable or the Tg *APP* allele might be on the same chromosome as *Chmp2b -* mouse chromosome 16 (Mmu16), preventing typical Mendelian segregation.

### Targeted locus Amplification analysis of J20 transgene insertion

**Figure 2.**
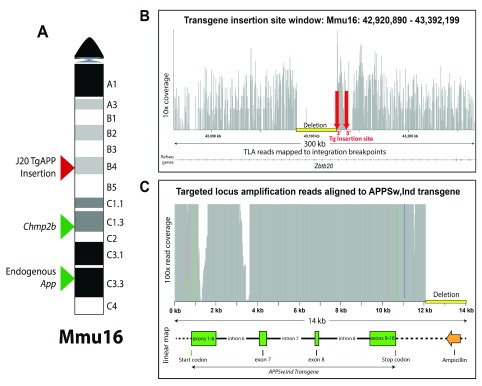
Insertion site of the J20 APP transgene (Tg) on mouse chromosome 16. ( **A**) Representation of Mmu16 showing insertion site of the J20 Tg array. Also shown are the positions of endogenous mouse *App* and *Chmp2b*. ( **B**) TLA applied to the APPSwInd transgene in the J20 mouse. Read mapping to the mouse (mm10) genome assembly shows the exact Tg insertion site (red arrows) with associated genomic deletion (yellow bar). Gene annotation is shown below labelling a small portion of intron 1 of the *Zbtb20* gene. ( **C**) TLA reads mapped to the APP Tg sequence. Complete coverage was achieved, showing that head to tail concatemerization of the Tg has occurred at position 12088 ablating the ampicillin cassette of the plasmid construct. Coloured lines represent SNPs found in the Tg sequence compared to the published sequence. A linearised map of the plasmid construct is shown below.

To determine the cause of the absence of *Chmp2b* ^-/-^;Tg *APP* ^J20^ offspring, we mapped the site of the J20 Tg *APP* transgene insertion, and so sequenced flanking regions around the insertion site by TLA. Sequence analysis ( Figure 2a) showed that the Tg *APP* insertion site lies on Mmu16. Targeted reads mapped the integration breakpoints to genomic co-ordinates Mmu16: 43,127,050 (3’ end of the transgene array) and Mmu16: 43,127,512 (5’ end of the transgene array). In addition, sequencing around the insertion site revealed a 41.17kb deletion in the mouse genome between chr16:43,085,979 and chr16:43,127,149 ( Figure 2b). Thus the lack of *Chmp2b* ^-/-^;Tg *APP* ^J20^ offspring is the result of the insertion of TgAPP ^J20^ on Mmu16, explaining the absence of *Chmp2b* ^-/-^;Tg *APP* ^J20^ progeny from the stage 2 cross.

TLA also allowed us to assess transgene sequence integrity. We found three SNPs (hAPP transgene sequence: 624 G > A, 979 G > A, 10649 G > A) and four indels (TG: 185 G > +1T, TG: 1168 G > +8GGCGGGAC, TG: 1423 C > +1G, TG: 5932 C > -1T) within the integrated transgene construct; however, all were silent mutations or found within intronic sequence. Furthermore, TLA analysis showed at least one transgene copy is truncated at the 3’ end (TG: 12088), ablating the ampicillin cassette, and is fused to the 5’ of another transgene copy to form a concatemer (TG:12088 fused to TG:3).

### Assessment of ZBTB20 expression

The integration site co-ordinates localize the J20 transgene insertion and deletion entirely within intron 1 of the gene Zinc-finger and BTB domain containing 20 *(Zbtb20,* transcript NM_001285805.1) on Mmu16. *Zbtb20* is a member of the BTB/POZ family of transcriptional repressors and functions primarily as a transcriptional repressor ( Xie *et al.*, 2008); it is important for hippocampal development and function, the site of greatest Aβ deposition in aging J20 animals ( Mucke *et al.*, 2000). Moreover, missense mutations in this gene are associated with Primrose syndrome, a cause of intellectual disability with autism ( Cordeddu *et al.*, 2014; Mattioli *et al.*, 2016). Additionally, haploinsufficiency of the gene has been suggested as an important factor in del3q13.31 syndrome, a cause of developmental delay and intellectual disability ( Rasmussen *et al.*, 2014). To determine whether transgene insertion has affected *Zbtb20* transcription in J20 animals in hippocampal tissue, we investigated mRNA and protein levels of ZBTB20 in wildtype and J20 animals.

**Figure 3.**
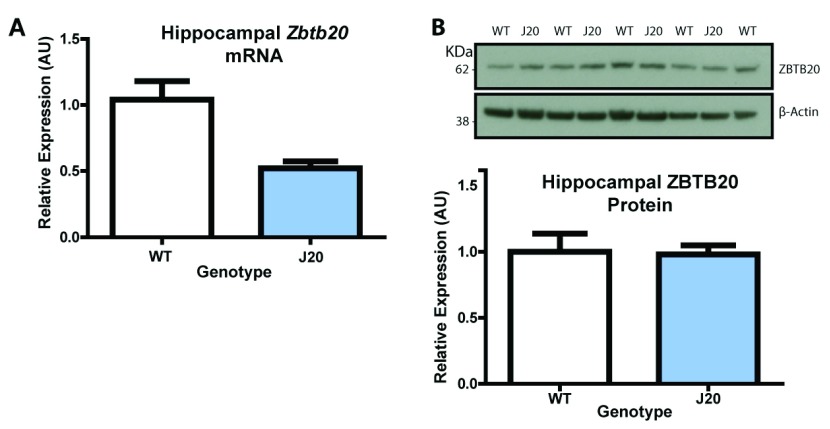
Expression of *Zbtb20* mRNA and protein in 3 month old wildtype and J20 ^+/-^ hippocampus. ( **A**) *Zbtb20* mRNA expression is significantly reduced in J20 hippocampus compared to wildtype (WT), detected by quantitative RT-PCR using primers spanning exons 8–9 (Mann Whitney U = 1, n = 5 wildtype/6 J20. P = 0.009). ( **B**) ZBTB20 protein levels are unchanged in J20 hippocampus compared to wildtype animals (Mann-Whitney U = 10, n = 5 wildtype/4 J20, P = 1). Sample sizes were too low to test for normality so non-parametric testing was conducted. Error bars show standard error of the mean; uncropped blots can be viewed at http://doi.org/10.17605/OSF.IO/4UGZF.

Firstly, a predesigned RT-qPCR assay was chosen from IDT to overlap the exon 8–9 boundary within the protein coding region of the *Zbtb20* transcript, downstream of the transgene insertion site. Importantly this junction is present in all predicted RefSeq protein coding transcript isoforms. We detected significantly less transcript in J20 animals compared to wildtype using this assay ( Figure 3a). To determine whether reduction in *Zbtb20* transcript results in a reduction of ZBTB20 protein in J20 animals, we assayed total hippocampal protein by western blot for ZBTB20 with an affinity purified polyclonal antibody, and observed that no change in ZBTB20 protein level in adult J20 hippocampal tissue could be determined by this method. We aimed to validate this result with another antibody (ab48889, Abcam) however, we were unable to determine a specific band at the correct molecular weight (data not shown). Because of the postnatal lethality demonstrated in *Zbtb20* ^-/-^ mice ( Sutherland *et al.*, 2009) and the complexity in producing control adult Zbtb20 ^-/-^ hippocampal tissue, we were not able to validate this result using an appropriate negative control. 

## Discussion

Using TLA we have located the insertion site of the J20 *APP* transgene on Mmu16 within intron 1 of the *Zbtb20* gene. We have also found a 41kb deletion of intronic sequence flanking the insertion site. Due to the key role of *Zbtb20* in hippocampal cell differentiation and function, we determined its expression in hippocampal tissue, and found that while *Zbtb20* transcript expression is reduced in J20 hippocampus, we could find no evidence that protein expression is altered, although we note the current technical limitations of our study because of the lack of an appropriate negative control. In a similar study investigating the transgene insertion site of the R6/2 Huntingdon’s disease model mouse, Jacobsen *et al.* discovered that the *HTT* exon 1 transgene inserted within intron 7 of the *Gm12695* gene, causing an almost 30 fold increase of expression of this gene compared to wildtype in cortical tissue irrespective of CAG-repeat length polymorphisms, showing that the genomic aberration caused by foreign DNA insertion within intronic sequence can alter gene regulation ( Jacobsen *et al.*, 2017). Notably, disruption of the expression of nearby genes may also occur in gene-targeted systems. For example, two of the four lines of mice that are null for the Prion protein gene *Prnp*, exhibited late onset ataxia and neurodegeneration -- this was subsequently found to be the result of aberrant upregulation of a gene ( *Prnd*) downstream of *Prnp*, which occurred as a result of induced exon skipping ( Moore *et al.*, 1999). In the J20 model, hippocampal *Zbtb20* transcription is perturbed without concomitant protein reduction. This is perhaps not as surprising as it seems; recent data indicate mRNA levels explain only around 40% of variability in protein levels ( Wilhelm *et al.*, 2014), with protein abundance being primarily dependent on translational control ( Schwanhäusser *et al.*, 2011; Schwanhäusser *et al.*, 2013).

It may be important to assess expression in multiple neuronal cell types and other tissues throughout development in the J20 model, considering that ectopic expression of *Zbtb20* in the subiculum and post-subiculum results in aberrant CA1 type development in those regions with associated CA1-specific markers ( Nielsen *et al.*, 2010) and the gene has been shown to have a role in liver function ( Sutherland *et al.*, 2009). The transgene sequence itself is intact in the J20, excluding several single nucleotide mutations; however, these are either silent or found within the transgene construct’s three intronic regions and are unlikely to affect the J20 transgene. TLA analysis and in-house copy number qPCR (data not shown) indicate that this transgene has integrated multiple times in an array on Mmu16. Multiple transgene insertion may be liable to recombination events, causing loss of some copies of the transgene, resulting in delayed onset of phenotype in affected animals. This potential confound can be allayed by undertaking a genomic copy-number qPCR to confirm copy-number consistency between individuals. The Jackson Laboratory have published their assay for general use.

Genetic engineering can result in unintended disruption of the genome, which can confound interpretation of phenotype if the genomic alterations cause changes in protein expression. Transgenic models have been and continue to be powerful tools for biomedical research, but knowledge of the insertion site and the local effects on gene expression will inform phenotyping studies.

## Data availability

Aligned BAM files from the TLA, uncropped western blot for Figure 3B, hippocampal qPCR data and western blot data for Zbtb20 are available on OSF http://doi.org/10.17605/OSF.IO/4UGZF ( Tosh, 2017).

## References

[ref-1] Bishop JO Smith P: Mechanism of chromosomal integration of microinjected DNA. *Mol Biol Med.* 1989;6(4):283–298. 2695741

[ref-2] Blake JA Eppig JT Kadin JA: Mouse Genome Database (MGD)-2017: community knowledge resource for the laboratory mouse. *Nucleic Acids Res.* 2017;45(D1):D723–D729. 10.1093/nar/gkw1040 27899570 PMC5210536

[ref-3] Cordeddu V Redeker B Stellacci E: Mutations in *ZBTB20* cause Primrose syndrome. *Nat Genet.* 2014;46(8):815–817. 10.1038/ng.3035 25017102

[ref-4] de Vree PJ de Wit E Yilmaz M: Targeted sequencing by proximity ligation for comprehensive variant detection and local haplotyping. *Nat Biotechnol.* 2014;32(10):1–9. 10.1038/nbt.2959 25129690

[ref-5] Games D Adams D Alessandrini R: Alzheimer-type neuropathology in transgenic mice overexpressing V717F beta-amyloid precursor protein. *Nature.* 1995;373(6514):523–527. 10.1038/373523a0 7845465

[ref-6] Ghazi-Noori S Froud KE Mizielinska S: Progressive neuronal inclusion formation and axonal degeneration in *CHMP2B* mutant transgenic mice. *Brain.* 2012;135(Pt 3):819–832. 10.1093/brain/aws006 22366797

[ref-7] Harris JA Devidze N Halabisky B: Many neuronal and behavioral impairments in transgenic mouse models of Alzheimer’s disease are independent of caspase cleavage of the amyloid precursor protein. *J Neurosci.* 2010;30(1):372–381. 10.1523/JNEUROSCI.5341-09.2010 20053918 PMC3064502

[ref-8] Haruyama N Cho A Kulkarni AB: Overview: engineering transgenic constructs and mice. *Curr Protoc Cell Biol.*(Hoboken, NJ, USA: John Wiley & Sons, Inc.),2009;Chapter 19:Unit 19.10. 10.1002/0471143030.cb1910s42 19283728 PMC2743315

[ref-9] Hong S Beja-Glasser VF Nfonoyim BM: Complement and microglia mediate early synapse loss in Alzheimer mouse models. *Science.* 2016;352(6286):712–716. 10.1126/science.aad8373 27033548 PMC5094372

[ref-10] Hooli BV Kovacs-Vajna ZM Mullin K: Rare autosomal copy number variations in early-onset familial Alzheimer’s disease. *Mol Psychiatry.* 2014;19(6):676–681. 10.1038/mp.2013.77 23752245

[ref-11] Jacobsen JC Erdin S Chiang C: Potential molecular consequences of transgene integration: The R6/2 mouse example. *Sci Rep.* 2017;7: 41120. 10.1038/srep41120 28120936 PMC5264158

[ref-12] Mattioli F Piton A Gérard B: Novel *de novo* mutations in *ZBTB20* in Primrose syndrome with congenital hypothyroidism. *Am J Med Genet Part A.* 2016;170(6):1626–1629. 10.1002/ajmg.a.37645 27061120

[ref-33] Moore RC Lee IY Silverman GL: Ataxia in prion protein (PrP)-deficient mice is associated with upregulation of the novel PrP-like protein doppel. *J Mol Biol.* 1999;292(4):797–817. 10.1006/jmbi.1999.3108 10525406

[ref-13] Mucke L Masliah E Yu GQ: High-level neuronal expression of abeta _1-42_ in wild-type human amyloid protein precursor transgenic mice: synaptotoxicity without plaque formation. *J Neurosci.* 2000;20(11):4050–4058. 10.1523/JNEUROSCI.20-11-04050.2000 10818140 PMC6772621

[ref-14] Mullan M Crawford F Axelman K: A pathogenic mutation for probable Alzheimer’s disease in the APP gene at the N-terminus of beta-amyloid. *Nat Genet.* 1992;1(5):345–347. 10.1038/ng0892-345 1302033

[ref-15] Murrell J Farlow M Ghetti B: A mutation in the amyloid precursor protein associated with hereditary Alzheimer’s disease. *Science.* 1991;254(5028):97–99. 10.1126/science.1925564 1925564

[ref-16] Nielsen JV Blom JB Noraberg J: Zbtb20-Induced CA1 Pyramidal Neuron Development and Area Enlargement in the Cerebral Midline Cortex of Mice. *Cereb Cortex.* 2010;20(8):1904–1914. 10.1093/cercor/bhp261 19955470

[ref-17] Palop JJ Mucke L: Network abnormalities and interneuron dysfunction in Alzheimer disease. *Nat Rev Neurosci.* 2016;17(12):777–792. 10.1038/nrn.2016.141 27829687 PMC8162106

[ref-18] Palop JJ Chin J Roberson ED: Aberrant excitatory neuronal activity and compensatory remodeling of inhibitory hippocampal circuits in mouse models of Alzheimer’s disease. *Neuron.* 2007;55(5):697–711. 10.1016/j.neuron.2007.07.025 17785178 PMC8055171

[ref-19] Rasmussen MB Nielsen JV Lourenço CM: Neurodevelopmental disorders associated with dosage imbalance of *ZBTB20* correlate with the morbidity spectrum of ZBTB20 candidate target genes. *J Med Genet.* 2014;51(9):605–613. 10.1136/jmedgenet-2014-102535 25062845

[ref-20] Roberson ED Scearce-Levie K Palop JJ: Reducing endogenous tau ameliorates amyloid beta-induced deficits in an Alzheimer’s disease mouse model. *Science.* 2007;316(5825):750–754. 10.1126/science.1141736 17478722

[ref-21] Robinson JT Thorvaldsdóttir H Winckler W: Integrative genomics viewer. *Nat Biotechnol.* 2011;29(1):24–26. 10.1038/nbt.1754 21221095 PMC3346182

[ref-22] Rockenstein EM McConlogue L Tan H: Levels and alternative splicing of amyloid beta protein precursor (APP) transcripts in brains of APP transgenic mice and humans with Alzheimer’s disease. *J Biol Chem.* 1995;270(47):28257–28267. 10.1074/jbc.270.47.28257 7499323

[ref-23] Sanchez PE Zhu L Verret L: Levetiracetam suppresses neuronal network dysfunction and reverses synaptic and cognitive deficits in an Alzheimer’s disease model. *Proc Natl Acad Sci U S A.* 2012;109(42):E2895–903. 10.1073/pnas.1121081109 22869752 PMC3479491

[ref-24] Sasahara M Fries JW Raines EW: PDGF B-chain in neurons of the central nervous system, posterior pituitary, and in a transgenic model. *Cell.* 1991;64(1):217–227. 10.1016/0092-8674(91)90223-L 1986868

[ref-25] Schwanhäusser B Busse D Li N: Global quantification of mammalian gene expression control. *Nature.* 2011;473(7374):337–342. 10.1038/nature10098 21593866

[ref-26] Schwanhäusser B Busse D Li N: Corrigendum: Global quantification of mammalian gene expression control. *Nature.* 2013;495(7439):126–127. 10.1038/nature11848 23407496

[ref-27] Skibinski G Parkinson NJ Brown JM: Mutations in the endosomal ESCRTIII-complex subunit CHMP2B in frontotemporal dementia. *Nat Genet.* 2005;37(8):806–808. 10.1038/ng1609 16041373

[ref-34] Sutherland AP Zhang H Zhang Y: Zinc finger protein Zbtb20 is essential for postnatal survival and glucose homeostasis. *Mol Cell Biol.* 2009;29(10):2804–2815. 10.1128/MCB.01667-08 19273596 PMC2682054

[ref-28] Tosh JL: J20 Transgene Mapping.2017 10.17605/OSF.IO/4UGZF

[ref-29] Truett GE Heeger P Mynatt RL: Preparation of PCR-quality mouse genomic DNA with hot sodium hydroxide and tris (HotSHOT). *BioTechniques.* 2000;29(1):52,54. 10.2144/00291bm09 10907076

[ref-30] Wilhelm M Schlegl J Hahne H: Mass-spectrometry-based draft of the human proteome. *Nature.* 2014;509(7502):582–587. 10.1038/nature13319 24870543

[ref-31] Xie Z Zhang H Tsai W: Zinc finger protein ZBTB20 is a key repressor of alpha-fetoprotein gene transcription in liver. *Proc Natl Acad Sci U S A.* 2008;105(31):10859–10864. 10.1073/pnas.0800647105 18669658 PMC2504784

[ref-32] Yan BW Zhao YF Cao WG: Mechanism of random integration of foreign DNA in transgenic mice. *Transgenic Res.* 2013;22(5):983–992. 10.1007/s11248-013-9701-z 23483296

