# Supplementary material for: The integration site of the APP transgene in the J20 mouse model of Alzheimer’s disease
Source: Wellcome Open Res. 2018 Oct 10;2:84. Originally published 2017 Sep 13. [Version 2] doi: 10.12688/wellcomeopenres.12237.2 (PMC5645710; doi:10.12688/wellcomeopenres.12237.2)
Supplement: Peer review history

# Peer review: Referee response for version 2

**DOI:** 10.21956/wellcomeopenres.16188.r34050

**Published:** 2018-11-28

**Authors:** Guillaume Pavlovic

**Affiliations:** 1 Institut Clinique de la Souris (ICS), CNRS, INSERM, University of Strasbourg, Illkirch, France; 2 PHENOMIN-ICS, Strasbourg, France

In this second version, the authors have responded very clearly and precisely to the three main remarks made by the reviewers.

I have read this submission. I believe that I have an appropriate level of expertise to confirm that it is of an acceptable scientific standard.

# Peer review: Referee response for version 1

**DOI:** 10.21956/wellcomeopenres.13248.r26484

**Published:** 2017-10-16

**Authors:** Guillaume Pavlovic

**Affiliations:** 1 Institut Clinique de la Souris (ICS), CNRS, INSERM, University of Strasbourg, Illkirch, France; 2 PHENOMIN-ICS, Strasbourg, France

The authors have found non mendalian ratio in cross between Chrmp2b-/- and J20+/- animals. They found that J20 transgene is integrated ~22 Mb of the Chrmp2b gene in the Zbtb20 gene.

 The report is well written, easy to understand and relevant. However, the Western Blot data does not allow to conclude that *Zbtb20* protein expression remains unchanged.

 Comments:

 - Figure 3

 Unpaired t-test: your statistical analysis hypothesize normal distribution of the data. Using a non parametric test will be more correct if you do not provide the normality test results.

 Please indicate what are the error bars (SEM, SD or 95% Cl)?

 - Assessment of ZBTB20 expression: Western Blot analysis

 The western blot results provided in the paper do not prove that the “ZBTB20 protein is unaltered in adult J20 hippocampal tissue”. Knock-out controls and molecular size markers are missing to prove that the showed band is not an unspecific signal.

 Moreover, the lack of appropriate controls (i.e. different quantity of protein extract) do not allow to validate the use of Figure 2B as a quantitative Western Blot. See why in the publications 1 to 3

 Finally, as recommended in PLOS ONE for example ( http://journals.plos.org/plosone/s/submission-guidelines), the author should provide:

- Original uncropped and unadjusted blots and gels, including molecular size markers, should be provided in either the figures or the supplementary files.
- The image should include all relevant controls, and controls should be run on the same blot or gel as the samples.
- All blots and gels that support results reported in the manuscript should be provided. Please provide ZBTB20 additional antibody results (which are data not shown in the paper) as additional files. Add reference of this additional antibody in material and methods.

I have read this submission. I believe that I have an appropriate level of expertise to confirm that it is of an acceptable scientific standard, however I have significant reservations, as outlined above.

## References

[1] TaylorSCPoschA: The design of a quantitative western blot experiment. *Biomed Res Int*.2014;2014: 10.1155/2014/361590 361590 10.1155/2014/361590 24738055PMC3971489

[2] TaylorSCBerkelmanTYadavGHammondM: A defined methodology for reliable quantification of Western blot data. *Mol Biotechnol*.2013;55(3) : 10.1007/s12033-013-9672-6 217-26 10.1007/s12033-013-9672-6 23709336PMC3840294

[3] GhoshRGildaJEGomesAV: The necessity of and strategies for improving confidence in the accuracy of western blots. *Expert Rev Proteomics*.2014;11(5) : 10.1586/14789450.2014.939635 549-60 10.1586/14789450.2014.939635 25059473PMC4791038

# Response

**Published:** 2018-09-23

**Authors:** Justin Tosh

**Affiliations:** University College London, Institute of Neurology, UK

The authors thank Dr Pavlovic for his insightful comments.

We have updated the figure legend to reflect the reviewers comments (error bars are SEM) and  have also sign-posted the link to the supplementary data in the legend for Figure 3; where all primary data including uncropped blots can be viewed, and note that the analysis of our western blots complied with best practice ( http://journals.plos.org/plosone/s/submission-guidelines).

In our qPCR and western blot experiments we could not test for, or assume normality of data distribution, so an alternative non-parametric test was used instead (Mann-Whitney U).

# Peer review: Referee response for version 1

**DOI:** 10.21956/wellcomeopenres.13248.r25970

**Published:** 2017-09-25

**Authors:** Takaomi C. Saido

**Affiliations:** 1 Laboratory for Proteolytic Neuroscience, RIKEN Brain Science Institute, Saitama, Japan

Gene expression is regulated by promoters, enhancers, silencers, etc. in a tissue-specific manner. For instance, Zbtb20 plays an essential role in hepatic *de novo* lipogenesis, so it is necessary to examine the mRNA and protein levels of Zbtb20 in all the tissues in addition to hippocampus including the liver because the transgene is inserted into every corresponding chromosome. In particular, it is surprising that the authors avoided examining developed and developing cortex because Zbtb20 modulates the sequential generation of neuronal layers in developing cortex. It would also be quite convincing if there was a negative control in Figure 3B (and in additional Western blot data) using tissues from Zbtb20-KO mice. One comment on the Western blot data, the quantities of protein are not sufficently normalized.

I have read this submission. I believe that I have an appropriate level of expertise to confirm that it is of an acceptable scientific standard, however I have significant reservations, as outlined above.

## References

[1] TonchevABTuocTCRosenthalEHStuderMStoykovaA: Zbtb20 modulates the sequential generation of neuronal layers in developing cortex. *Mol Brain*.2016;9(1) : 10.1186/s13041-016-0242-2 65 10.1186/s13041-016-0242-2 27282384PMC4901408

[2] LiuGZhouLZhangHChenRZhangYLiLLuJYJiangHLiuDQiSJiangYMYinKXieZShiYLiuYCaoXChenYXZouDZhangWJ: Regulation of hepatic lipogenesis by the zinc finger protein Zbtb20. *Nat Commun*.2017;8: 10.1038/ncomms14824 14824 10.1038/ncomms14824 28327662PMC5364431

# Response

**Published:** 2018-09-23

**Authors:** Justin Tosh

**Affiliations:** University College London, Institute of Neurology, UK

The authors thank Dr Saido for his insightful comments.

We agree the transgene is inserted into *Zbtb20* in every cell of the body, and therefore may affect multiple organs. However it is beyond the scope of this initial characterization of the genomic location of the *APP *transgene in the J20 animal model to study transcription of *Zbtb20* in every organ of the mouse. We wished to communicate the issue of disruption of the *Zbtb20* locus to the scientific community immediately so that further detailed studies can be undertaken.  

We note that have not been able to source tissue from either Zbtb20 ^-/-^ or Zbtb20 ^-/+^ mice, and that Zbtb20 ^-/-^ mice have poor postnatal survival (Sutherland 2009). Thus it is not possible at this time to verify the results presented in Figure 3B using an appropriate negative control (adult hippocampus from a Zbtb20 ^-/-^). Therefore we agree that until we can undertake this important validation of the experiment, we cannot state categorically that the ZBTB20 protein level is unchanged in the J20 model system and have edited our paper accordingly.

# Peer review: Referee response for version 1

**DOI:** 10.21956/wellcomeopenres.13248.r25971

**Published:** 2017-09-19

**Authors:** Karen H. Ashe

**Affiliations:** 1 Department of Neurology, University of Minnesota, Minneapolis, MN, 55455, USA

The authors have shown that an unexpected ratio of genotypes that occurred when J20 mice were bred to Chmp2b KO mice was due to the insertion of the J20 transgene array near the Chmp2b gene, resultig in linkage disequilibrium. The report is interesting, relevant and well-done. The only correction I recommend is to change Figure 1 so that the colors in the rectangles match the colors of the mice (e.g., make the orange rectangle red and the red rectangle orange).

I have read this submission. I believe that I have an appropriate level of expertise to confirm that it is of an acceptable scientific standard.

# Response

**Published:** 2018-09-23

**Authors:** Justin Tosh

**Affiliations:** University College London, Institute of Neurology, UK

The authors thank Professor Ashe for her insightful comments. In Version 2 of the article we have altered Figure 1 to improve colour clarity as suggested.
